# Microbiome Dysregulation and Inflammation: Key Players in Pulmonary Hypertension Pathophysiology

**DOI:** 10.3390/biomedicines13112750

**Published:** 2025-11-11

**Authors:** Lan Zhao

**Affiliations:** Department of Medicine, Division of Pulmonary, Allergy and Critical Care Medicine, Stanford University, Stanford, CA 94305, USA; lanzhao20140101@gmail.com

Pulmonary hypertension (PH) is a fatal disease characterized by elevated pulmonary pressures, progressive pulmonary vascular remodeling, and right heart failure [1,2]. The burgeoning field of microbiome research has witnessed significant advancements in recent years, driven by technological innovations and collaborative research efforts [3,4]. Researchers have characterized gut microbial communities by utilizing state-of-the-art sequencing technologies, such as 16S rRNA gene sequencing and shotgun metagenomics, across multiple PH human and animal cohorts [5,6,7,8]. Growing evidence suggests that gut microbial changes contribute to PH through modulation of immune and metabolic pathways, connecting the gut–lung axis to PH pathogenesis [5,7]. The review paper titled “Pulmonary Hypertension and the Gut Microbiome” [9] provides a comprehensive summary of available experimental and clinical evidence, linking gut microbiome imbalances to the development and progression of PH, particularly the pulmonary arterial hypertension (PAH). The review describes how alterations in gut microbiome composition, including reduced microbial diversity, depletion of anti-inflammatory short-chain fatty acid (SCFA)-producing bacteria, and enrichment of proinflammatory species may impair gut barrier integrity, allowing for translocation of microbial metabolic products, such as lipopolysaccharide (LPS) and trimethylamine N-oxide (TMAO) into circulation. These microbial metabolites activate toll-like receptor 4 (TLR4) signaling, inflammatory cytokine release, and pathways involving serotonin and arginine metabolism, all of which contribute to endothelial dysfunction and pulmonary vascular remodeling in PH [10,11,12]. Moreover, human and animal studies reveal distinct gut microbiome profiles that predict PH presence and severity, with elevated microbial TMAO correlating with worse clinical outcomes [12,13]. The review also highlights emerging therapeutic targets, including probiotics, prebiotics, dietary modification, and fecal microbiota transplantation, as potential adjuncts to current vasodilator treatments for PH [14,15,16].

Besides the above paper in the Special Issue, which emphasized the role of serotonin in determining health outcomes, another review paper [17] from Prof. Cassidy Delaney’s group explored the multifaceted roles of serotonin signaling in normal lung development and in the pathogenesis of neonatal PH. Serotonin (5-hydroxytryptamine, 5-HT) is mainly synthesized in the gut, and the gut microbiome significantly influences serotonin production [18,19]. Serotonin acts as both a neurotransmitter and a vasoactive mediator that regulates pulmonary vascular tone and smooth muscle proliferation in the developing lung and PAH pathogenesis [12,17,18]. Serotonin contributes to PH through activation of 5-HT receptors (especially 5-HT_1_B/5-HT_2_A) and uptake via the serotonin transporter (SERT), leading to marked vasoconstriction and elevated pulmonary vascular resistance [18]. Elevated serotonin levels or enhanced SERT activity increase pulmonary vascular resistance, stimulate vascular remodeling, and contribute to persistent pulmonary hypertension of the newborn (PPHN), a life-threatening condition associated with intracardiac shunting and hypoxemia. The authors reviewed studies implicating maternal factors, such as selective serotonin reuptake inhibitor use and hypoxia, in altering serotonin homeostasis, which, in turn, exacerbates neonatal PH risk [20,21,22]. They also highlight that enhanced serotonin production, receptor activation, and transporter-mediated uptake converge to drive the vasoconstrictive and proliferative phenotype characteristic of PH [23,24], making the serotonin pathway an attractive therapeutic target. Overall, the review underscores serotonin as a critical developmental regulator and a promising target for preventing or treating neonatal PH.

A review of chronic thromboembolic pulmonary hypertension (CTEPH) pathobiology [25] and a case report about PAH-related spontaneous bacterial peritonitis (SBP) [26] were also included in the Special Issue. CTEPH, a rare but potentially surgical curable form of PH, arises when unresolved thrombi become fibrotic and obstruct pulmonary arteries, leading to pulmonary embolism (PE) and eventual right heart failure [27]. The review [25] highlights the interplay of multiple pathological processes, such as prothrombotic state, fibrinolysis resistance, defective angiogenesis, and chronic inflammation, on CTEPH risk [28,29,30]. Importantly, genetic factors, such as variants in fibrinogen [31] and ABO blood group [32], as well as contributors, including gut microbiome dysbiosis, low-grade infections, circulating microparticles, and plasma metabolic disturbances affecting lipid- and amino acid-signaling pathways, are all known to be associated with the pathobiology of CTEPH [12,33,34,35]. Despite these advances, the review emphasized that many pathways overlap with acute PE and venous thromboembolism, leaving the question of why only a minority of PE patients develop CTEPH unresolved. The authors conclude that integrating genetic, epigenetic, microbiome, and metabolomic research will be critical for guiding future preventive and therapeutic strategies for CTEPH. Although ascites is typically caused by cirrhosis, cardiac ascites accounts for ~3% of cases and rarely progresses to SBP due to the preserved opsonic and bactericidal activity of its protein-rich fluid [36,37,38]. This case report [26] describes a 75-year-old man with severe PAH who developed new-onset ascites complicated by SBP—a rare manifestation of right heart failure [39]. The patient presented with dyspnea and abdominal distension. Diagnostic paracentesis revealed a serum–ascites albumin gradient (SAAG) ≥1.1 g/dL and high protein content consistent with cardiac ascites [40,41], while a polymorphic nucleated cell count (PMN) of 663 cells/mm3 confirmed SBP [36,42]. Imaging and clinical data supported decompensated right heart failure secondary to PAH rather than liver cirrhosis. He was successfully treated with antibiotics, diuretics, and fluid restriction. This unique case underscores that PAH-related right heart failure can lead to portal hypertension, congestive hepatopathy, and susceptibility to SBP despite the traditionally low infection risk of cardiac ascites, and it calls for the development of management guidelines specific to SBP in non-cirrhotic ascites.

Finally, a research study [43] in the Special Issue explored the regulatory role and mechanism of N6-methyladenosine (m6A) RNA modification in PAH. m6A is the most abundant internal RNA modification in eukaryotic cells, and emerging evidence [44,45] shows that it plays a key regulatory role in PH by controlling gene expression and vascular remodeling. Using monocrotaline (MCT)-induced PAH rats, the authors performed methylated RNA immunoprecipitation sequencing (MeRIP-seq) and RNA-seq to map transcriptome-wide m6A changes and associated gene expression in pulmonary artery tissues. They identified 209 genes with significantly altered m6A peaks, revealing widespread hypermethylation and hypomethylation enriched in extracellular matrix (ECM) remodeling, MAPK, and PI3K/AKT signaling pathways, which are central to PAH pathogenesis [46,47,48]. Integration of m6A and transcriptomic data highlighted 42 key genes with concurrent changes in methylation and expression, among which Centromere Protein F (*Cenpf*) emerged as a hub gene that promotes pulmonary artery smooth muscle cell (PASMC) proliferation. The study also discovered a novel m6A reader, Leucine-Rich Pentatricopeptide Repeat Containing (*Lrpprc*), which was significantly downregulated in PAH. Further experiments showed that silencing *Lrpprc* enhanced PASMC proliferation and increased *Cenpf* expression, suggesting that *Lrpprc* negatively regulates vascular remodeling through m6A-dependent mechanisms. Overall, the findings provide a comprehensive map of m6A modifications in PAH pulmonary arteries, implicating dysregulated m6A signaling in abnormal PASMC growth and identifying potential epigenetic targets for therapeutic intervention. Although the work does not directly investigate the microbiome, the identified genes are closely connected to inflammatory processes that drive pulmonary vascular inflammation and remodeling in PAH.

In summary, the assembled reviews [9,17,25] in the Special Issue highlight how gut microbiome alterations, including loss of beneficial taxa and expansion of proinflammatory microbes, can elevate circulating metabolites (e.g., LPS, TMAO) and activate serotonin, TLR4, and other signaling pathways to modulate the immune/inflammatory responses that promote pulmonary vascular remodeling and PH progression. The case report found that PAH-related SBP [26], although rare, can be lethal if not properly treated with antibiotics and other treatments. The original study [43] demonstrates widespread m6A modifications in PAH pulmonary arteries, particularly in genes linked to vascular remodeling, inflammation, and cell proliferation, thereby revealing novel epigenetic targets for PH. Altogether, the collection proposes that the gut microbiota interacts with the lungs through the gut–lung axis, which is mediated through microbial metabolism and inflammatory signaling. While the gut microbiome has received considerable attention due to its profound impact on host physiology and health, recent studies have extended the investigation to various anatomical niches, including the upper airway [49,50] and lungs [7,8], which may impact PH pathogenesis and host response. An important outlook for the field is that microbiome and inflammation research is reframing PH as a systemic, immune–metabolic disease rather than an isolated pulmonary vascular disorder. Thus, microbial dysbiosis, inflammation, and intersection with molecular modifications in vascular cells are emerging as crucial and interlinked contributors to PH pathogenesis and attractive therapeutic targets.
